# P-556. Mind the Gap: Assessing College Students’ Awareness of Vaccination Status for Mandated Vaccines

**DOI:** 10.1093/ofid/ofaf695.771

**Published:** 2026-01-11

**Authors:** Nicole Bradley, Yuman Lee

**Affiliations:** St. John's University, Queens, New York; St. John's University, Queens, New York

## Abstract

**Background:**

Amidst the measles outbreak, awareness of immunization status against vaccine preventable diseases (VPD) is crucial, especially on college campuses. Per the World Health Organization, herd immunity against the measles requires about 95% of the population to be vaccinated. In New York State (NYS), college students born after 1957 must provide proof of immunity against measles, mumps, and rubella (MMR) and complete a meningococcal meningitis vaccination response form. The purpose of this study was to determine college students’ awareness of their vaccination status against state mandated vaccination requirements.

**Methods:**

An anonymous electronic survey was distributed to students who attended a university-wide health fair in April 2025 in Queens, NY. Sex, birth year, affiliated college, and assessment of student reported vaccination status against MMR and meningococcal meningitis were collected.

**Results:**

Of the 86 students who responded, majority (98.8%) were born in 2000 or later, and over half (57%) were students in the College of Pharmacy and Health Sciences. Approximately 13% of students reported they were unvaccinated or unsure of their vaccination status against MMR. Similarly, 11.6% of students reported they were unvaccinated or unsure of their vaccination status against meningococcal meningitis.

**Conclusion:**

Results from this study demonstrate the need for increased education and outreach on VPD among college students. The 11-13% of students who reported being unvaccinated or unsure about their status for mandated vaccines reveal a significant gap in vaccine awareness, rather than a true lack of vaccination. This disconnect highlights issues in vaccine literacy and raises public health concerns such as vaccine hesitancy, threats in herd immunity, and delayed responses during outbreaks. Targeting educational initiatives can improve students’ understanding of VPD and ensure they are better prepared to respond to current and future public health threats.

**Disclosures:**

All Authors: No reported disclosures

